# Supplementary material for: Disruption of day‐to‐night changes in circadian gene expression with chronic tendinopathy
Source: J Physiol. 2023 Mar 9;602(23):6509–24. doi: 10.1113/JP284083 (PMC11607887; doi:10.1113/JP284083)
Supplement: Supplementary file 8 — Supporting information [file TJP-602-6509-s008.pdf]

Disruption of day-to-night changes in circadian gene expression with chronic tendinopathy

Ching-Yan Chloé Yeung[1,2]*, René B Svensson[1,2], Kateryna Yurchenko[1,2], Nikolaj M. Malmgaard-Clausen[1,2], Ida Tryggedsson[1,2], Marius Lendal[1,2], Anja Jokipii-Utzon[1,2], Jens L Olesen[1,2], Yinhui Lu[3], Karl E Kadler[3], Peter Schjerling[1,2], Michael Kjær[1,2]

1. Institute of Sports Medicine Copenhagen, Department of Orthopedic Surgery, Copenhagen University Hospital – Bispebjerg and Frederiksberg, Copenhagen, Denmark.
2. Center for Healthy Aging, Department of Clinical Medicine, University of Copenhagen, Denmark.
3. Wellcome Centre for Cell-Matrix Research, Faculty of Biology, Medicine and Health, University of Manchester, Manchester, UK.

Supplementary Figures 1-5 (PDF)

Supplementary Table 1 (PDF)

Supplementary Data 1-6 (Excel)

Data file S1. Original data used for generating statistics. (Excel)

Data file S2. Statistical analysis output for healthy tendon RNAseq data. (Excel)

Data file S3. Enrichment analysis of time-dependent RNAs of healthy tendon. (Excel)

Data file S4. Raw data output from automated fibril diameter measurements. (Excel)

Data file S5. Statistical analysis output for chronic tendinopathy RNAseq data. (Excel)

Yeung et al. – Figure S1

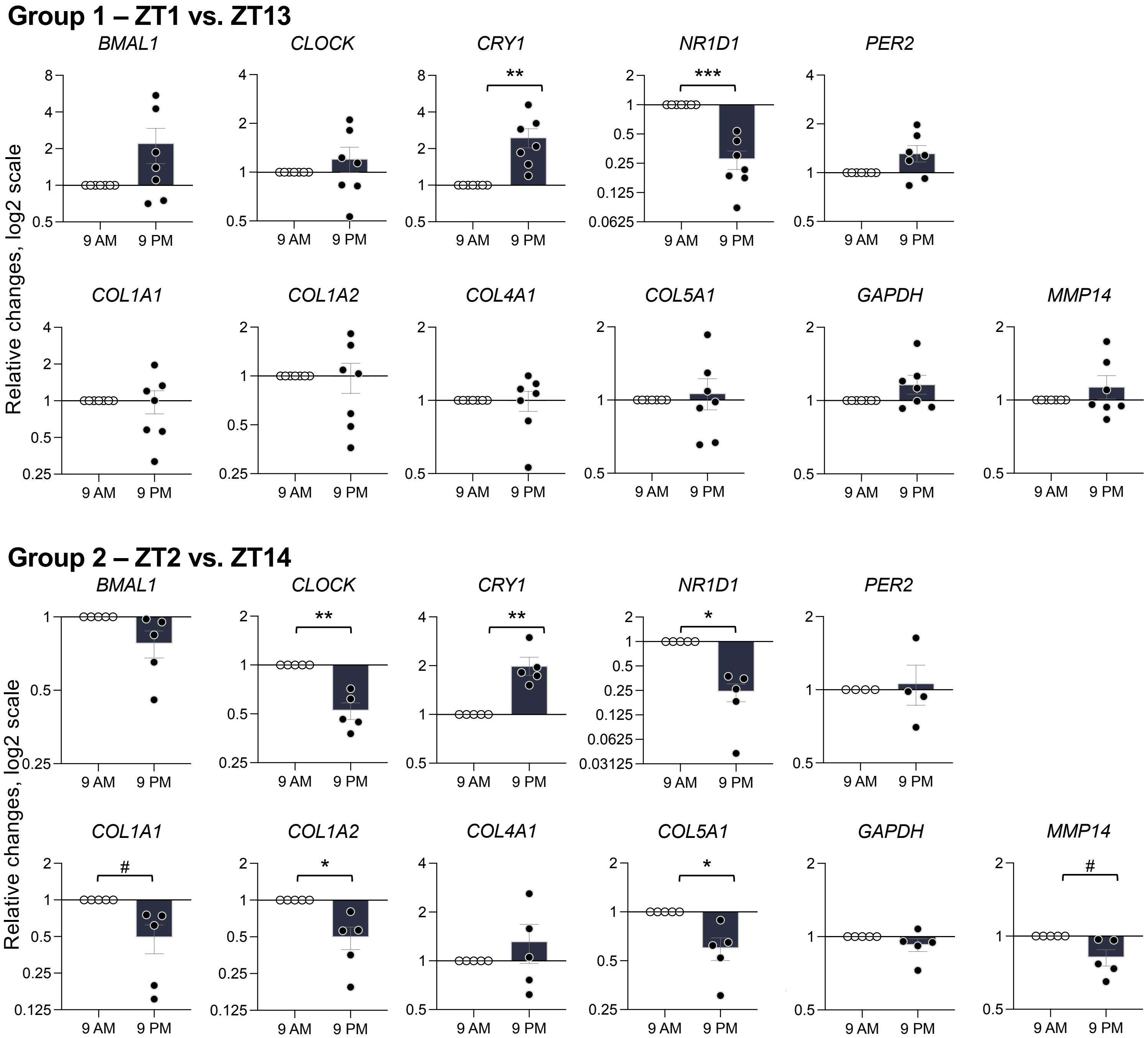

Fig. S1. Statical output for comparison of gene expression in human tendon biopsies according to ZT. Human patellar tendon biopsies were taken at two time points in a day, at 9 AM and 9 PM. The expression of circadian clock genes and collagen genes were analysed by RT-qPCR. Expression was normalised to RPLP0, and expressed in log2 values, relative to the day biopsy value. Group 1 (n = 7): **P = 0.0038 (*CRY1*), ***P = 0.0008 (*NR1D1*). Group 2 (n = 5, except n = 4 for *PER2*): **P = 0.0044 (*CLOCK*), **P = 0.0044 (*NR1D1*), *P = 0.0146 (*PER2*), $^{\#}P$ = 0.573 (*COL1A1*), *P = 0.0301 (*COL1A2*), *P = 0.0310 (*COL5A1*), $^{\#}P$ = 0.0514 (*MMP14*), from paired t-tests performed on log-transformed data. Values are means ± SEM. N = 4 for *PER2* in Group 2 due to failed PCRs in one 9 AM and one 9 PM sample.

Yeung et al. – Figure S2

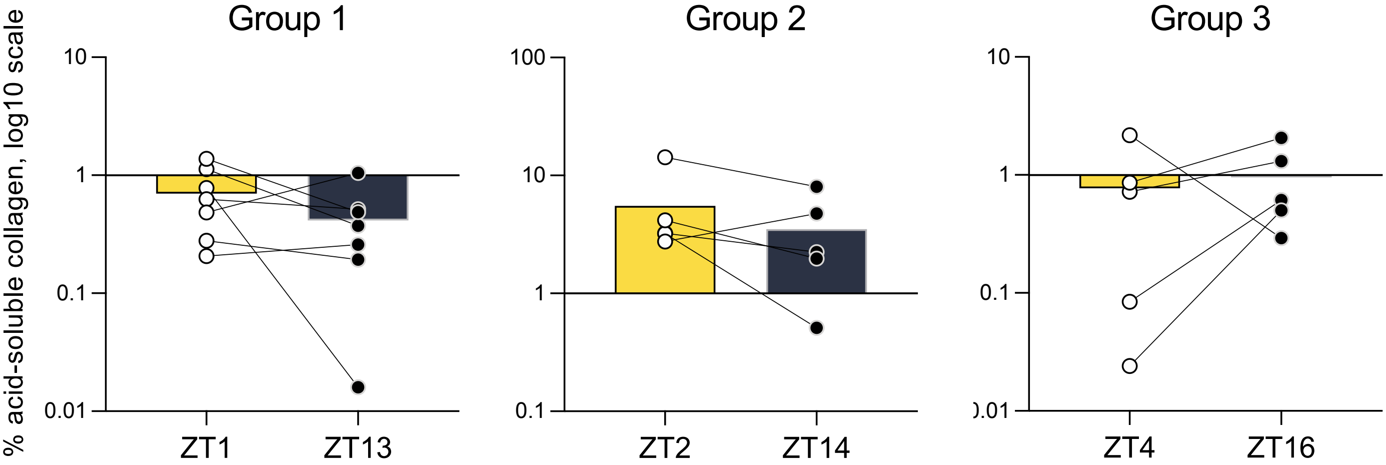

Fig. S2. Comparison of the acid-soluble collagen fraction in human tendon biopsies according to ZT.
The percentage of acid-soluble collagen was determined in patellar tendon biopsies taken at 9 AM and 9 PM. Statistical analysis showed no significant difference between the amount of acid-soluble collagen in tendon tissues taken in the morning and the night when analysed in groups based on similar ZT: Group 1: ZT1, ZT13 (n = 7), Group 2: ZT2, ZT14 (n = 5) and Group 3: ZT4, ZT16 (n = 5) (bars show mean; P = 0.2118, 0.1935 and 0.3484, respectively, from paired t-tests on log-transformed data).

Yeung et al. – Figure S3

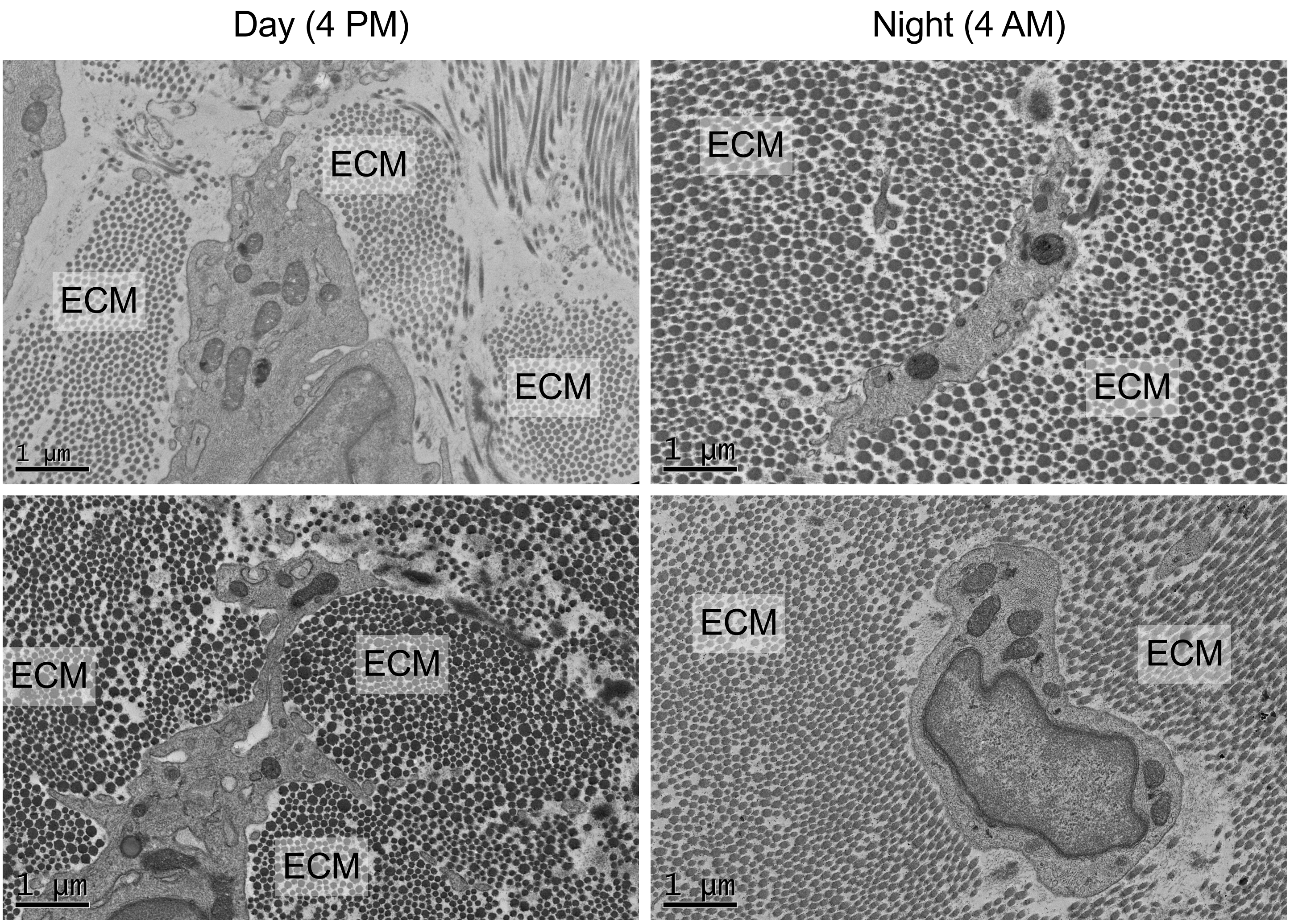

Fig. S3. Representative TEM images of patellar tendon biopsies.
Images of transverse sections of two different patellar tendon biopsy samples taken during the day (4 PM) and two different samples taken during the night (4 AM). Electron-dense circles are collagen fibrils (ECM). Bars = 1 µm.

Yeung et al. – Figure S4

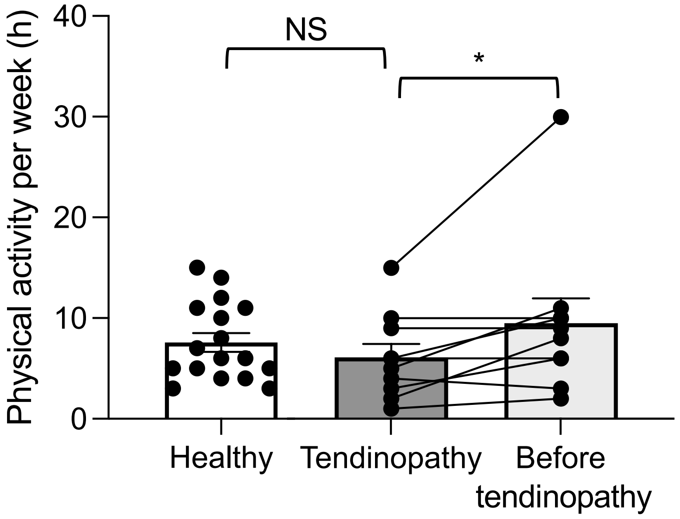

Fig. S4. Levels of physical activity in study participants.
There was no significant difference between the number of hours spent on physical activity per week at the time of sampling between the healthy and tendinopathic participants (n = 17 (healthy), n = 10 (tendinopathy); bars show mean; P = 0.3589, unpaired t-test). However, participants with chronic tendinopathy had significantly more hours of physical activity per week before they were affected (n = 10; bars show mean; P = 0.0469, Wilcoxon matched-pairs signed rank test (n = 10).

Yeung et al. – Figure S5

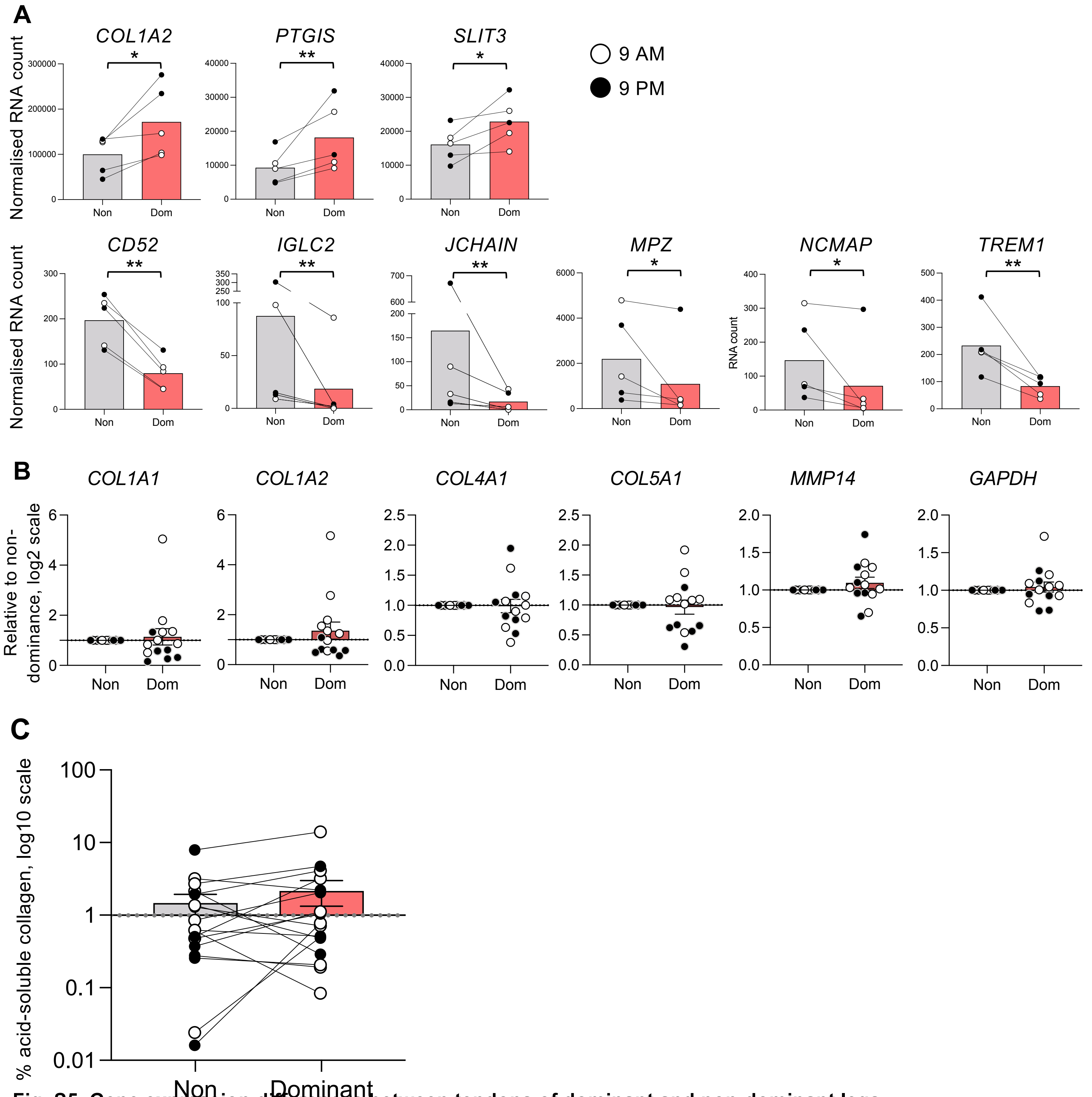

Fig. S5. Gene expression differences between tendons of dominant and non-dominant legs.
Statistical analysis between gene expression in patellar tendon biopsies of the dominant leg (Dom) and non-dominant (Non) leg showed 9 differentially regulated genes. **(A)** Normalised RNA counts for *COL1A2*, *PTGIS* and *SLIT3* were significantly upregulated (log2 fold change 0.6 to 1.0) in tendon biopsies of the dominant leg (*n* = 5 biological samples; *P* (FDR)= 0.0447, 0.0062, 0.0447, respectively; from DESeq2 analysis). RNA counts for *CD52, IGLC2, JCHAIN, MPZ, NCMAP* and *TREM1* were significantly upregulated (log2 fold change 1.1 to 1.4) in tendon biopsies of the non-dominant leg (*n* = 5 biological samples; *P* (FDR) = 0.0062, 0.0062, 0.0062, 0.0447, 0.0447, 0.0078, respectively; from DESeq2 analysis). Although significant, *IGCL2*, *JCHAIN* and *NCMAP* had log2 fold change of -1^{e-5} (shrunken values) making a real change questionable. Connecting lines indicate paired samples. **(B)** Changes in gene expression relative to the dominant biopsy value (Dom) in all the healthy biopsies analysed by RT-qPCR. Expression was normalised to RPLP0 and expressed in log2 values (*n* = 17; bars show SEM; *P* > 0.05, from paired t-tests performed on the log-transformed data). **(C)** The percentage of acid-soluble collagen was determined in patellar tendon biopsies taken at 9 AM and 9 PM. Statistical analysis showed no significant difference between the amount of acid-soluble collagen in tendon tissues taken from the dominant or the non-dominant (Non) leg (*n* = 17 biological samples; bars show mean; *P* = 0.3174, from paired t-test performed on log-transformed data).

Yeung et al. – Table S1

	Age (y)	Wall time of day biopsy (hh:mm)	Wall time of night biopsy (hh:mm)	ZT of day biopsy (hours)	ZT of night biopsy (hours)	*N*
**HEALTHY CONTROLS**						
For RNAseq						
All biopsies used	24.4 ± 2.3	09:02 ± 00:07	20:54 ± 00:08	3.1 ± 1.6	15.0 ± 1.6	5, 5
- Batch 1 (Group 3)	25.7 ± 1.5	09:07 ± 00:06	20:57 ± 00:03	4.3 ± 0.5	16.1 ± 0.5	3, 3
- Batch 2 (Group 2)	22.5 ± 2.1	08:57 ± 00:04	20:49 ± 00:13	1.4 ± 0.2	13.2 ± 0.4	2, 2
For RT-qPCR analyses						
All biopsies used	24.6 ± 3.1	09:09 ± 00:11	21:05 ± 00:13	1.5 ± 1.3	13.5 ± 1.3	14, 14
- Group 1: ZT1 vs ZT13	25.4 ± 2.9	09:13 ± 00:11	21:05 ± 00:13	0.7 ± 0.2	12.6 ± 0.2	7, 7
- Group 2: ZT2 vs ZT14	25.0 ± 2.9	09:05 ± 00:10	21:06 ± 00:17	1.6 ± 0.2	13.6 ± 0.4	5, 5
For collagen content assays						
All biopsies used	24.8 ± 2.9	09:09 ± 00:10	21:03 ± 00:12	2.0 ± 1.6	13.9 ± 1.6	17, 17
- Group 1: ZT1 vs ZT13	25.4 ± 2.9	09:13 ± 00:11	21:05 ± 00:13	0.7 ± 0.2	12.6 ± 0.2	7, 7
- Group 2: ZT2 vs ZT14	25.0 ± 2.9	09:05 ± 00:10	21:06 ± 00:17	1.6 ± 0.2	13.6 ± 0.4	5, 5
- Group 3: ZT4 vs ZT16	23.6 ± 3.0	09:07 ± 00:10	20:59 ± 00:04	4.3 ± 0.4	16.2 ± 0.4	5, 5
For TEM						
All biopsies used	24.6 ± 2.1 (day), 24.9 ± 2.2 (night)	16:20 ± 00:18	04:07 ± 00:07	10.6 ± 0.2	22.4 ± 0.1	9, 8
**CHRONIC TENDINOPATHY**						
For RNAseq						
All biopsies used	38.3 ± 13.7 (day), 26.3 ± 5.2 (night)	08:54 ± 00:13	21:04 ± 00:18	0.7 ± 0.4	12.6 ± 0.4	5, 4
For RT-qPCR analyses						
All biopsies used	35.4 ± 13.5 (day), 27.4 ± 5.2 (night)	08:53 ± 00:13	21:00 ± 00:17	0.7 ± 0.4	12.6 ± 0.4	5, 5
Contralateral biopsies	35.4 ± 13.5 (day), 27.4 ± 5.2 (night)	09:02 ± 00:11	21:08 ± 00:17	0.6 ± 0.5	12.6 ± 0.4	5, 5

Table S1. Tendon biopsy collection times as a function of wall time and zeitgeber (ZT) time.
All values are mean ± SD. Human patellar tendon biopsies were taken at two time points in a day, 12 hours apart. For RNAseq, RT-qPCR and acid-soluble collagen fraction analyses biopsies were taken at 9 AM and 9 PM during different times of the year as described in the Materials and Methods. For TEM analysis, biopsies were taken at 4 PM and then at 4 AM as described in the Materials and Methods. ZT, zeitgeber time. Values are mean + SD.
